# Protosappanin A protects against pathological cardiac hypertrophy by inhibiting oxidative stress and NLRP3 inflammasome-mediated pyroptosis via activation of the Nrf2 signaling pathway

**DOI:** 10.3389/fcvm.2025.1682641

**Published:** 2026-01-09

**Authors:** Qing He, Yun Zheng, Xiaoli Yan, Shanshan Lv, Bo Yu

**Affiliations:** Department of Cardiovascular Surgery, Xijing Hospital, Air Force Medical University, Xi’an, Shaanxi, China

**Keywords:** cardiac hypertrophy, Nrf2 signaling pathway, oxidative stress, protosappanin A, pyroptosis

## Abstract

**Introduction:**

Pathological cardiac hypertrophy is a pivotal pathological process underlying various cardiac diseases, including heart failure (HF). Protosappanin A (PTA), a major biphenyl compound isolated from *Caesalpinia sappan*, has been shown to confer significant protective effects against multiple cardiovascular insults. However, its precise role in pressure overload-induced pathological cardiac hypertrophy remains elusive.

**Methods:**

In the present study, a mouse model was established through transverse aortic constriction (TAC) surgery and then intragastrically administered with PTA for 4 weeks.

**Results:**

Our results indicate that PTA treatment led to an improvement in cardiac contractile function, a reduction in cardiomyocyte hypertrophy, and an attenuation of myocardial fibrosis in TAC-operated mice. Notably, PTA exerted its anti-hypertrophic actions by mitigating myocardial oxidative stress injury and inhibiting cardiomyocyte pyroptosis. Nevertheless, the above cardioprotective effects of PTA were largely abrogated by the use of the nuclear factor erythroid 2-related factor 2 (Nrf2) specific inhibitor ML385 in TAC-treated mice or Nrf2 siRNA in angiotensin II (Ang II)-treated neonatal mouse cardiomyocytes (NMCMs).

**Discussion:**

Our study demonstrates for the first time that PTA ameliorates cardiac remodeling and dysfunction in mice with pathological cardiac hypertrophy by suppressing oxidative stress and cardiomyocyte pyroptosis through activating of the Nrf2 signaling pathway, highlighting additional therapeutic option for clinical prevention and treatment of HF patients.

## 1 Introduction

Pathological cardiac hypertrophy induced by pressure overload is recognized as a crucial precursor and an independent risk factor for heart failure (HF) (1, 2). The sustained pathological stimuli, triggered by hypertension (3), myocardial infarction (4), or valvular dysfunction (5, 6), plays a pivotal role in the development of pathological cardiac hypertrophy, ultimately leading to irreversible damage to cardiac structure and function. Although current medical practices can alleviate the clinical symptoms of HF patients, they fail to halt the progression of cardiac hypertrophy. Moreover, the side effects of medications and the interactions between different drugs represent significant concerns in clinical management (7). Therefore, further exploration of the underlying mechanisms of pathological cardiac hypertrophy and the development of novel, safe, and effective therapeutic strategies are of paramount importance for delaying or even reversing cardiac hypertrophy and treating HF.

*Caesalpinia sappan*, a member of the legume family, has long been utilized as a traditional Chinese medicinal herb (8). The diverse array of chemical components present in this plant possesses a wide spectrum of biological activities, encompassing anti-inflammatory, antibacterial, antioxidant, antitumor, immunosuppressive, and vasodilatory properties, demonstrating high application value and broad prospects (9). Protosappanin A (PTA) is a major biphenyl compound isolated from *Caesalpinia sappan (*9). Previous studies have found that PTA exhibits remarkable anti-rejection effects in heart transplantation (10, 11). Importantly, recent investigations have revealed that PTA exerts a protective role in various cardiovascular disorders, including doxorubicin-induced cardiotoxicity (12), myocardial ischemia-reperfusion injury (12), autoimmune myocarditis (13), and atherosclerosis (14). However, the exact role of PTA in pathological cardiac hypertrophy as well as the underlying mechanisms remain ambiguous.

Oxidative stress injury stands as a central mechanism in the onset and progression of pathological cardiac hypertrophy (15). The imbalance between oxidative and antioxidant systems, primarily resulting from the excessive production of reactive oxygen species (ROS), not only induces cardiomyocyte hypertrophy and promotes myocardial fibrosis but also leads to cardiomyocyte death, collectively accelerating cardiac remodeling and the deterioration of cardiac function (16, 17). Additionally, numerous studies have demonstrated that ROS can facilitate the activation of the NLRP3 inflammasome through multiple mechanisms, subsequently causing cell membrane rupture and triggering pyroptosis (18, 19). As a pro-inflammatory form of cell death, pyroptosis impacts cardiac function by reducing the cardiomyocyte numbers, and also exacerbates inflammatory responses and myocardial injury by releasing inflammatory factors and cellular contents (20, 21). Therefore, inhibiting oxidative stress injury and cardiomyocyte pyroptosis represents a crucial strategy for the prevention and treatment of pathological cardiac hypertrophy.

Nuclear factor erythroid 2-related factor 2 (Nrf2) is a pivotal transcription factor in the cellular responses to oxidative stress, capable of regulating the expression of various downstream antioxidant enzymes, such as NAD(P)H quinone oxidoreductase 1 (NQO1) and heme oxygenase 1 (HO-1), to scavenge ROS and thereby enhance cellular antioxidant capacity (22, 23). Studies have confirmed that activation of the Nrf2 signaling pathway exhibits significant protective effects in a range of cardiovascular diseases, including myocardial infarction, myocardial ischemia-reperfusion injury, doxorubicin-induced cardiotoxicity, diabetic cardiomyopathy, and heart failure (24, 25). Furthermore, recent research has also reported that activation of the Nrf2 signaling pathway can suppress the activation of the NLRP3 inflammasome and pyroptosis by inhibiting ROS production (26). However, whether the Nrf2 signaling pathway could mediate the cardioprotective effects of PTA has not been investigated yet.

Herein, the primary objective of this study is to explore the impact of PTA on pressure overload-induced pathological cardiac hypertrophy and elucidate the underlying molecular mechanisms. Both TAC-induced mouse model and angiotensin II (Ang II)-induced cellular model revealed that PTA showed outstanding anti-hypertrophic effects by inhibiting oxidative stress and NLRP3 inflammasome-mediated pyroptosis in a manner of activating the Nrf2 signaling pathway. Collectively, the findings of our experiments shed new light on pharmacological approaches for the clinical prevention and treatment of HF patients.

## 2 Materials and methods

### 2.1 Reagents

Protosappanin A (PTA, HY-113573, 99.98%) and ML385 (HY-100523) were purchased from MedChemExpress (NJ, USA). Angiotensin II (Ang II) was purchased from Solarbio Biotechnology (A9290, Beijing, China). DHE (S0063) and DCFH-DA (S0036S) fluorescent probes were purchased from Beyotime Biotechnology (Shanghai, China). Superoxide dismutase (SOD, E-BC-K020-M), malondialdehyde (MDA, E-BC-K025-M), Interleukin 1 Beta (IL-1β, E-EL-M0037) and Interleukin 18 (IL-18, E-EL-M0730) assay kits were purchased from Elabscience Biotechnology (Wuhan, China). Lipofectamine 3000 was purchased from Thermo Fisher Scientific (L3000008, MA, USA). Propidium iodide (PI, S7109) solution and Bicinchoninic acid (BCA, BCA1) kit were purchased from Sigma-Aldrich (MO, USA). RNA extraction kit was purchased from TaKaRa Biomedical Technology (9767, Beijing, China). Keap1 (10503-2-AP), HO-1 (10701-1-AP, 1:1000), NLRP3 (68102-1-Ig, 1:1000), Histone H3 (17168-1-AP, 1:1000), and GAPDH (60004-1-Ig, 1:5000) antibodies were purchased from Proteintech (IL, USA). Nrf2 (#20733, 1:1000), ASC (#67824, 1:1000), cleaved caspase-1 (#89332, 1:1000), and GSDMD-N (#10137, 1:1000) antibodies were purchased from Cell Signaling Technology (MA, USA). NQO-1 (ab80588, 1:1000) antibody was purchased from Abcam (Cambridge, UK).

### 2.2 Animals and grouping

All animal procedures were approved by the Air Force Medical University Committee on Animal Care (Approval No. IACUC-20230761). Male C57BL/6 mice aged 6–8 weeks were purchased from the Experimental Animal Center of the Air Force Medical University, and were housed under standard conditions of care (22 °C ± 2 °C, and a 12 h light/dark cycle). Food and water were available *ad libitum*.

C57BL/6 mice were randomly assigned to the following groups: (1) Sham group: mice underwent the sham operation and were intragastrically administered with vehicle (10% DMSO diluted in sterile saline) for 4 weeks; (2) TAC group: mice underwent the TAC surgery and were intragastrically administered with vehicle for 4 weeks; (3) TAC + PTA group: mice underwent the TAC surgery and were intragastrically administered with PTA (10 mg/kg or 20 mg/kg) for 4 weeks; (4) TAC + PTA + ML385 group: mice underwent the TAC surgery and were intragastrically administered with PTA for 4 weeks. At the same time, ML385 (30 mg/kg) was given intraperitoneally one day before and once every three days after the TAC operation. PTA was dissolved in 10% DMSO and diluted in sterile saline.

### 2.3 Transverse aortic constriction (TAC) surgery

As described previously (27), a mouse model of cardiac hypertrophy was established by performing the TAC surgery. Briefly, mice were anesthetized with 2% isoflurane, followed by endotracheal intubation and connection to a small animal ventilator (RWD Life Science, Shenzhen, China). A 0.5-cm incision was made in the second intercostal space, and the muscle layers and thymus were bluntly dissected to expose the aortic arch. A suture was passed between the innominate artery and the left common carotid artery. Utilizing a 27-gauge needle as a spacer, the suture was tightened to constrict the aorta to the predetermined width. After withdrawing the needle to finalize the constriction, the sternal muscles and skin were sequentially sutured. The mice were placed on a warming pad for recovery and then transferred to a caging system for continuous observation. The sham-operated mice underwent the identical surgical procedure except for the ligation of the aortic arch.

### 2.4 Echocardiographic evaluation

The left ventricular systolic function and ventricular wall thickness of mice were assessed using the VisualSonics 2100 echocardiography system (VisualSonics, Toronto, Canada). In brief, after being anesthetized with 2% isoflurane, the mice were fixed in a supine position on the operating table. Ultrasound gel was evenly applied to the heart area, followed by the use of a 30 MHz ultrasound transducer to obtain the optimal cardiac ultrasound images. Vevo Lab 3.1.0 software (VisualSonics, Toronto, Canada) was utilized to quantify key parameters, including the left ventricular ejection fraction (LVEF), left ventricular fractional shortening (LVFS), interventricular septal thickness in diastole (IVSd), and left ventricular posterior wall thickness at end diastole (LVPWd).

### 2.5 Histological staining

Fresh myocardial tissue was fixed in 4% paraformaldehyde and then prepared into 5-μm thick paraffin sections. As described previously (4), Masson staining and WGA staining were employed to assess the degree of myocardial fibrosis and the mean cross-sectional area of cardiomyocytes, respectively, in order to evaluate the myocardial remodeling. For each heart section, five non-overlapping fields of view were randomly selected. In each field, the cross-sectional area of approximately 20 cardiomyocytes was measured using ImageJ software for statistical analysis.

### 2.6 Neonatal mouse cardiomyocytes (NMCMs) culture and treatment

Neonatal mice aged 1–3 days were disinfected using 75% ethanol. Hearts were promptly excised and then placed in pre-cooled PBS. The myocardial tissues were uniformly minced into small pieces of approximately 1 mm^3^ and subsequently transferred into centrifuge tubes containing digestive enzymes (collagenase and trypsin). The tissues were digested in a 37 °C incubator for 30 min, and this process was repeated three times until digestion was complete. The cell suspension was then filtered through a 70-μm filter, and the supernatant was discarded after centrifugation, with the cardiomyocytes collected from the precipitate. The cardiomyocytes were resuspended in complete medium, and purification was achieved through differential adhesion method, exploiting the difference in adhesion speed between fibroblasts and cardiomyocytes. Next, Ang II was applied to NMCMs at the concentration of 1 μM in the presence or absence of PTA (50 μM or 100 μM) intervention for 48 h.

### 2.7 Cell transfection

NMCMs were transfected with Nrf2 siRNA using Lipofectamine 3000 to achieve knockdown of the *Nfe2l2* gene. Nrf2 siRNA was synthesized by the GenePharma Company (Suzhou, China). The sequence of the Nrf2 siRNA were: sense: CCGAAUUACAGUGUCUUAATT, anti-sense: UUAAGACACUGUAAUUCGGTT. One day prior to transfection, cells were seeded into 6-well plates at a density of approximately 80% confluency. The cells were washed twice with PBS, followed by the addition of serum-free medium. Subsequently, siRNA and Lipofectamine 3000 were separately diluted with Opti-MEM (Thermo Fisher Scientific, MA, USA), gently mixed together, and incubated at room temperature for 20 min to form the siRNA transfection complex. The incubated complex was then evenly added to each well and cultured for 6 h. Afterwards, the serum-free medium was replaced with complete medium to terminate the transfection.

### 2.8 ROS detection

ROS production was detected in the myocardial tissue and in NMCMs using the DHE and DCFH-DA fluorescent probes, respectively.

Fresh myocardial tissue was rapidly frozen at −80 °C and then sectioned into 5-μm thick slices. After washing three times with PBS, the slices were incubated with 10 μM DHE staining solution at 37 °C in the dark for 30 min. Subsequently, the nuclei were stained with DAPI at room temperature for 15 min. After another wash with PBS, the slices were observed under a laser confocal microscope (FV3000, Olympus, Japan). Five non-adjacent fields per heart section were randomly selected, and the average fluorescence intensity was measured per field. The red fluorescence intensity was analyzed using ImageJ software (NIH, USA) to reflect the relative production of ROS.
NMCMs were seeded in confocal dishes at a density of approximately 80% confluency. Once the cells were grouped and treated, they were washed three times with serum-free medium, followed by the incubation with 1 mM DCFH-DA working solution at 37 °C in the dark for 20 min. After another wash with PBS, the cells were immediately observed under a laser confocal microscope. The green fluorescence intensity was analyzed using ImageJ software to reflect the relative production of ROS.

### 2.9 Determination of SOD activity and MDA content

The oxidative stress levels in the myocardial tissues and NMCMs were assessed using purchased SOD and MDA kits following the manufacturer's instructions. The data were analyzed spectrophotometrically using a SpectraMax M5 device (Molecular Devices, CA, USA) to calculate the SOD activity and MDA content.

### 2.10 Determination of IL-1β and IL-18 levels

Levels of IL-1β and IL-18 in the serum or supernatant medium were determined by the corresponding ELISA kits according to the manufacturer's instructions. The data were analyzed spectrophotometrically using a SpectraMax M5 device (Molecular Devices, CA, USA).

### 2.11 Immunofluorescence staining

Fresh myocardial tissue was fixed in 4% paraformaldehyde and then prepared into 5-μm thick paraffin sections. Antigen retrieval was performed on the sections using sodium citrate-EDTA solution, followed by blocking non-specific binding sites with 5% BSA. Diluted specific primary antibodies were applied to the sections and incubated overnight at 4 °C. The sections were washed three times with PBS, and then fluorescently labeled secondary antibodies were added and incubated at room temperature in the dark for 1 h. Finally, the nuclei were counterstained with DAPI, and the sections were observed and photographed under a fluorescence microscope (Nikon Eclipse C1, Nikon, Japan).

### 2.12 PI staining

NMCMs were seeded in confocal dishes at a density of approximately 80% confluency. After grouping and treatment, the cells were washed once with PBS. Subsequently, the cells were incubated with 1 ml of staining buffer containing Hoechst (5 µg/ml) and PI (2 µg/ml) at 4 °C for 30 min. Following another wash with PBS, PI-positive cells exhibiting red fluorescence were observed under a laser confocal microscope.

### 2.13 Quantitative real-time PCR

Fresh tissue or cellular samples were obtained, and total RNA was extracted using an RNA extraction kit. Subsequently, cDNA was synthesized utilizing reverse transcriptase, and the cDNA template, along with primers specific for the target genes, were mixed to prepare the qPCR reaction mixture. The CFX96 Real-Time System (Bio-Rad Laboratories, CA, USA) was then employed to perform amplification according to a preset program. The expression levels of the target genes were calculated using the 2^−ΔΔCT^ method and normalized to the standard gene (*Gapdh*). The sequences of primers used in this study were synthesized by Sangon Biotechnology (Shanghai, China) and are listed in Table 1.

**Table 1:** Sequences of primers used for qRT-PCR.

Gene	Forward (5′-3′)	Reverse (5′-3′)
*Anp*	GTGCGGTGTCCAACACAGAT	GCCATTTCCTCCGACTTTTCTC
*Bnp*	TGCCACCTTTTGACAGTGATG	TGATGTGCTGCTGCGAGATT
*Ctgf*	GCTGCCTACCGACTGGAAGAC	CCTAATGGCTTCCACCCTCTTC
*Col1a1*	GGAGACAGGTCAGACCTGTGTG	CAGCTGGATAGCGACATCGGC
*Col3a1*	ATGGTGGTTTTCAGTTCAGC	GCCTTGAATTCGCCTTCATT
*Nrf2*	GCTGTGACCTGTCACTGTGT	GCTGTGACCTGTCACTGTGT
*Gapdh*	AGAACATCATCCCTGCATCC	AGTTGCTG TTGAAGTCGC

### 2.14 Western blot

Proteins were extracted from tissue or cellular samples and subjected to quantitative analysis using the BCA assay. The protein samples were then loaded onto a polyacrylamide gel for electrophoresis separation, followed by transfer onto a polyvinylidene difluoride (PVDF) membrane. The membrane was blocked with 5% non-fat milk, and subsequently incubated with specific primary antibodies overnight at 4 °C. After washing with Tris-buffered saline containing Tween 20 (TBST) buffer, the membrane was incubated with the corresponding secondary antibodies at room temperature for 2 h. The expression of the target proteins was detected using enhanced chemiluminescence (ECL) reagent and the ChemiDoc™ XRS system (Bio-Rad Laboratories, CA, USA).

### 2.15 Statistical analysis

All data are presented as the mean ± standard error of the mean (SEM) and analyzed using GraphPad Prism 9.0 (GraphPad Software, CA, USA). The differences between multiple groups were compared by one-way ANOVA followed by Bonferroni correction for *post hoc t-*test. *P* < 0.05 were considered to be statistically significant. At least three independent experiments were conducted to ensure reproducibility.

## 3 Results

### 3.1 PTA improves cardiac systolic function in mice after TAC surgery by activating the Nrf2 signaling pathway

**Figure 1:**
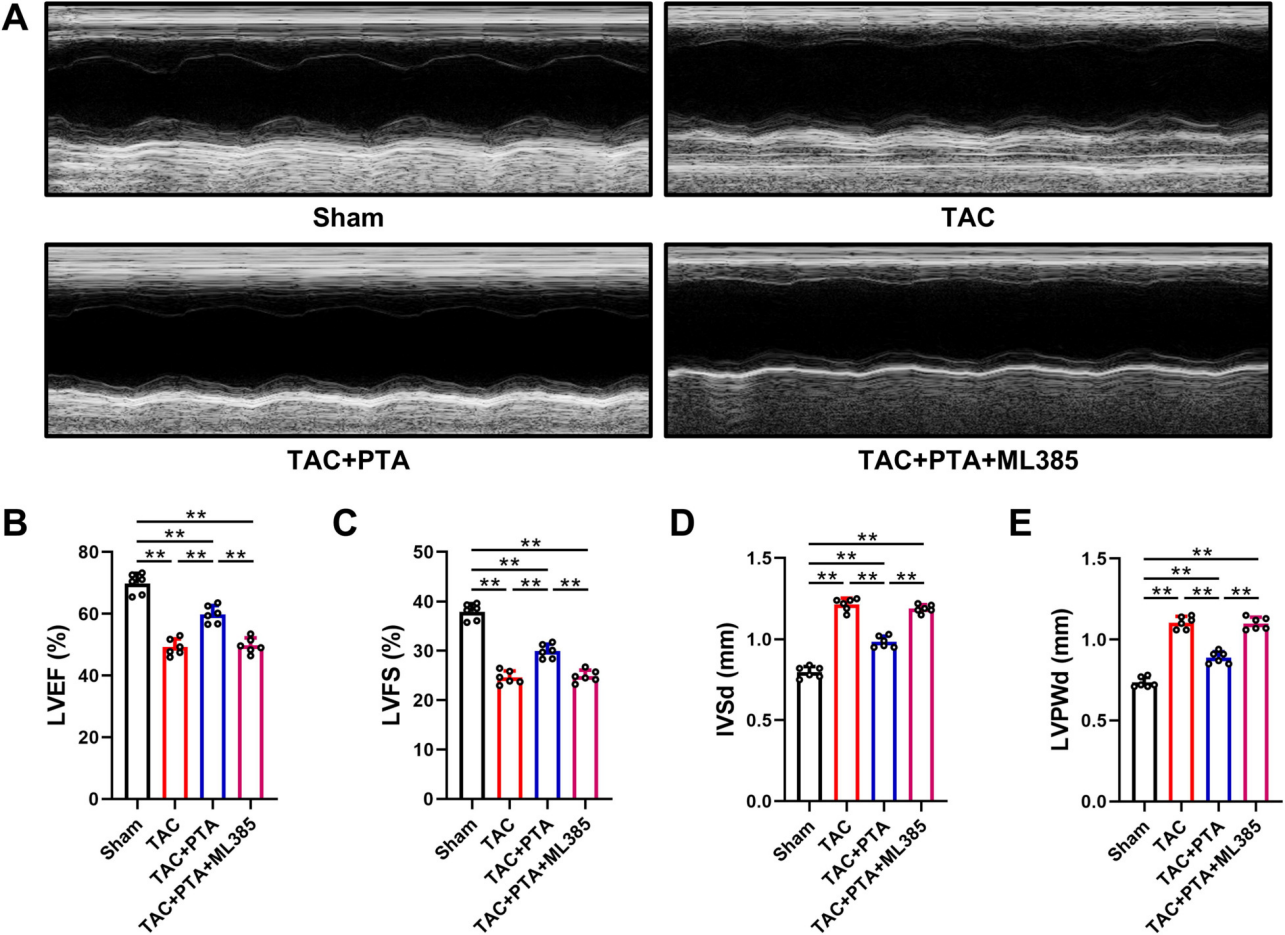
PTA improves cardiac systolic function in mice after TAC surgery by activating the Nrf2 signaling pathway. **(A–D)** Quantitative analysis of LVEF, LVFS, IVSd and LVPWd (*n* = 6 mice per group). PTA was administrated at a concentration of 20 mg/kg. **P < 0.01 between the two indicated groups. The data are presented as the mean ± SEM.

Representative M-mode echocardiographic images were acquired to evaluate cardiac function. As illustrated in Figures 1A,B, both LVEF and LVFS, which serve as indicators of cardiac systolic function, were reduced in the TAC group compared to the Sham group. Notably, treatment with PTA significantly enhanced LVEF and LVFS values in mice that underwent TAC surgery in a dose-dependent manner (Supplementary Figure S1). Therefore, in the subsequent *in vivo* experiments, we administered PTA at a concentration of 20 mg/kg for the intervention. It is worth mentioning that the administration of ML385, a specific inhibitor of Nrf2, markedly attenuated the protective effects of PTA on cardiac systolic dysfunction induced by TAC, when compared with the TAC + PTA group.

### 3.2 PTA ameliorates cardiac remodeling in mice after TAC surgery by activating the Nrf2 signaling pathway

**Figure 2:**
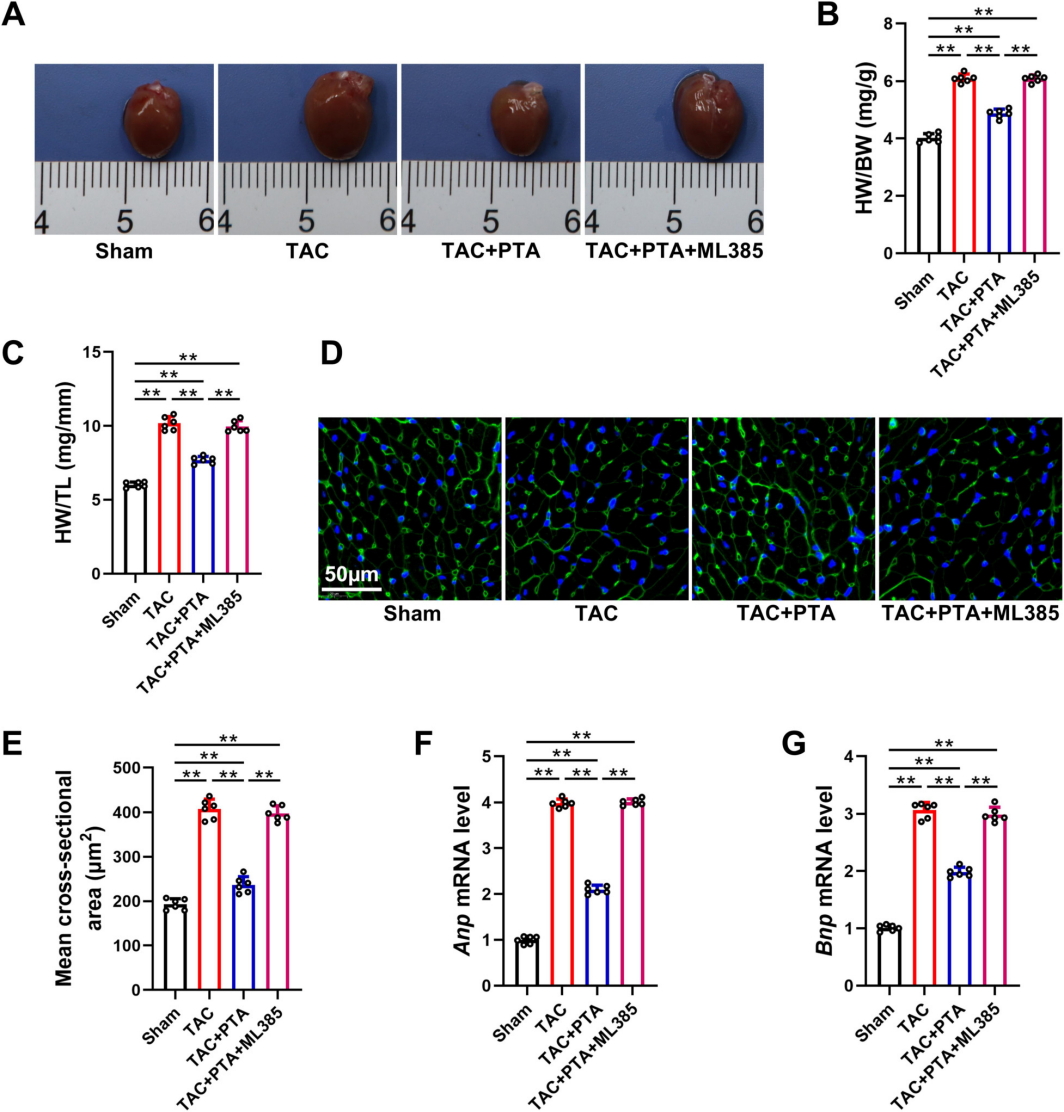
PTA ameliorates cardiac hypertrophy in mice after TAC surgery by activating the Nrf2 signaling pathway. **(A)** Representative images of the whole hearts. **(B)** The HW/BW ratio (*n* = 6 mice per group). **(C)** The HW/TL ratio (*n* = 6 mice per group). **(D,E)** Representative images of WGA staining and quantitative analysis of mean cross-sectional area of cardiomyocyte (*n* = 6 mice per group, scale bar = 50 μm). **(F,G)** qRT-PCR analysis of *Anp* and *Bnp* mRNA levels in the heart tissues (*n* = 6 mice per group). PTA was administrated at a concentration of 20 mg/kg. **P < 0.01 between the two indicated groups. The data are presented as the mean ± SEM.

Cardiac hypertrophy represents a prominent structural alteration in myocardial remodeling triggered by TAC (28). As depicted in Figures 1C,D, echocardiographic evaluation also demonstrated a statistically significant augmentation in the thickness of both the interventricular septum and the left ventricular posterior wall in the TAC group compared to the Sham group, primarily reflected in the LVSd and LVPWd parameters. Additionally, the heart size, the heart weight to body weight ratio (HW/BW) and heart weight to tibia length ratio (HW/TL) were notably elevated in mice subjected to TAC surgery (Figures 2A–C). As shown in Figures 2D,E, WGA staining disclosed a substantial increase in the mean cross-sectional area of cardiomyocytes following TAC surgery. Furthermore, the mRNA expression levels of two classic cardiac hypertrophic markers, atrial natriuretic peptide (ANP) and brain natriuretic peptide (BNP), were upregulated in response to TAC operation as well (Figures 2F,G). Conversely, a marked attenuation of cardiac hypertrophy was evident in these aforementioned indices subsequent to treatment with PTA. However, the anti-hypertrophic effects elicited by PTA were abrogated by the co-administration of ML385, when compared to the TAC + PTA group.

**Figure 3:**
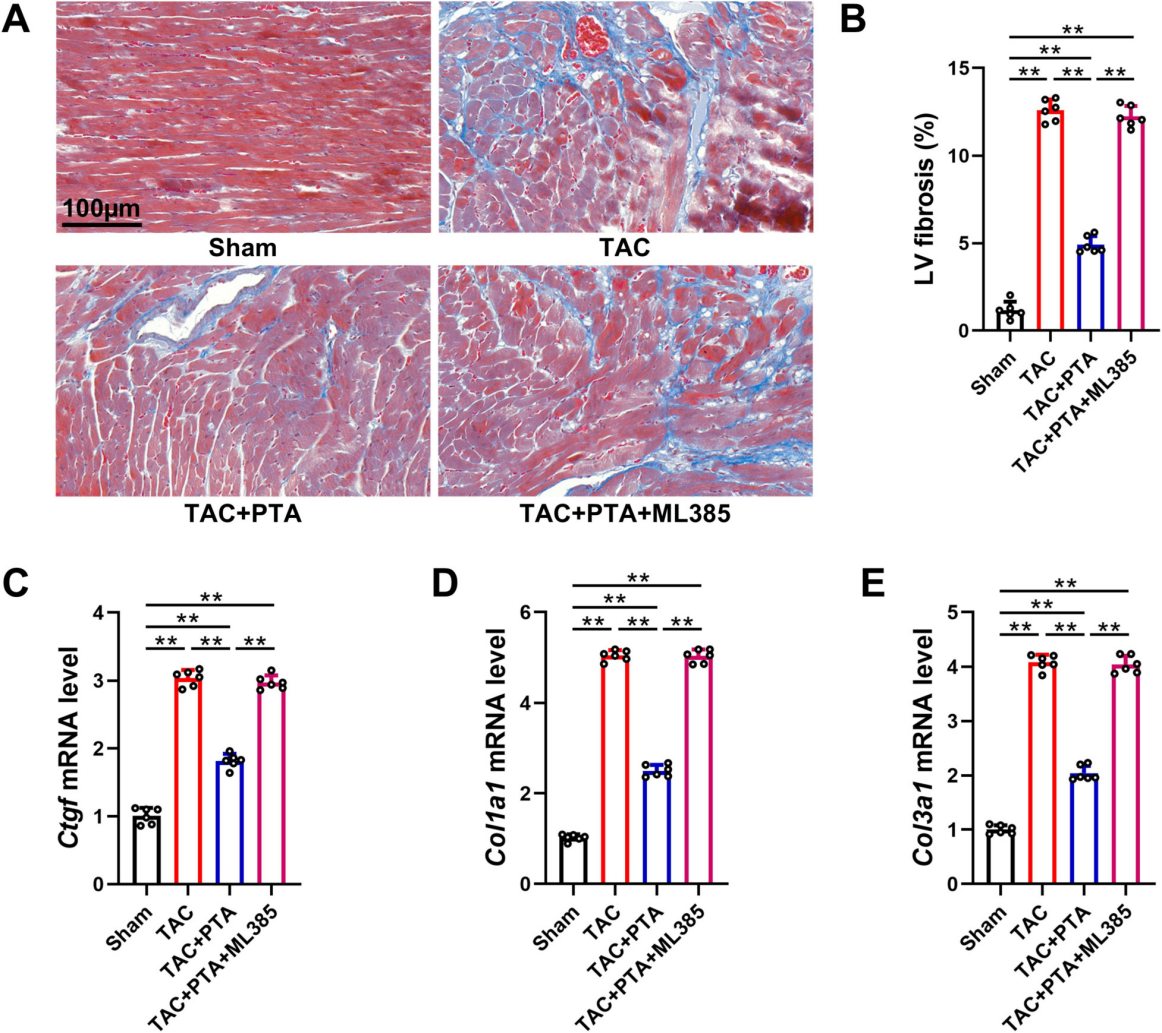
PTA ameliorates myocardial fibrosis in mice after TAC surgery by activating the Nrf2 signaling pathway. **(A,B)** Representative images of Masson staining and semi-quantitative analysis of interstitial cardiac fibrosis (*n* = 6 mice per group, scale bar = 100 μm). **(C–E)** qRT-PCR analysis of *Ctgf*, *Col1a1* and *Col3a1* mRNA levels in the heart tissues (*n* = 6 mice per group). PTA was administrated at a concentration of 20 mg/kg. **P < 0.01 between the two indicated groups. The data are presented as the mean ± SEM.

Myocardial fibrosis is another pivotal characteristic of TAC-caused cardiac remodeling (29). As illustrated in Figures 3A,B, a notable increase in collagen deposition was observed within the myocardial tissues of mice at 4 weeks post-TAC surgery. Additionally, PCR analysis revealed that the profibrotic response was significantly enhanced following TAC operation, corroborated by the upregulation of several fibrosis-associated genes, including connective tissue growth factor (CTGF), Collagen 1 (Col1a1) and Collagen 3 (Col3a1) (Figures 3C–E). Remarkably, compared to the TAC group, PTA treatment mitigated collagen accumulation and reduced the expression levels of CTGF, Col1a1, and Col3a1. Similarly, ML385 administration substantially blunted the protective effects of PTA against TAC-induced myocardial fibrosis. Taken together, the above findings suggest that PTA exhibits inhibitory efficacy on TAC-induced cardiac remodeling, and this effect is predominantly dependent on the Nrf2 signaling pathway.

### 3.3 PTA mitigates myocardial oxidative stress in mice after TAC surgery by activating the Nrf2 signaling pathway

**Figure 4:**
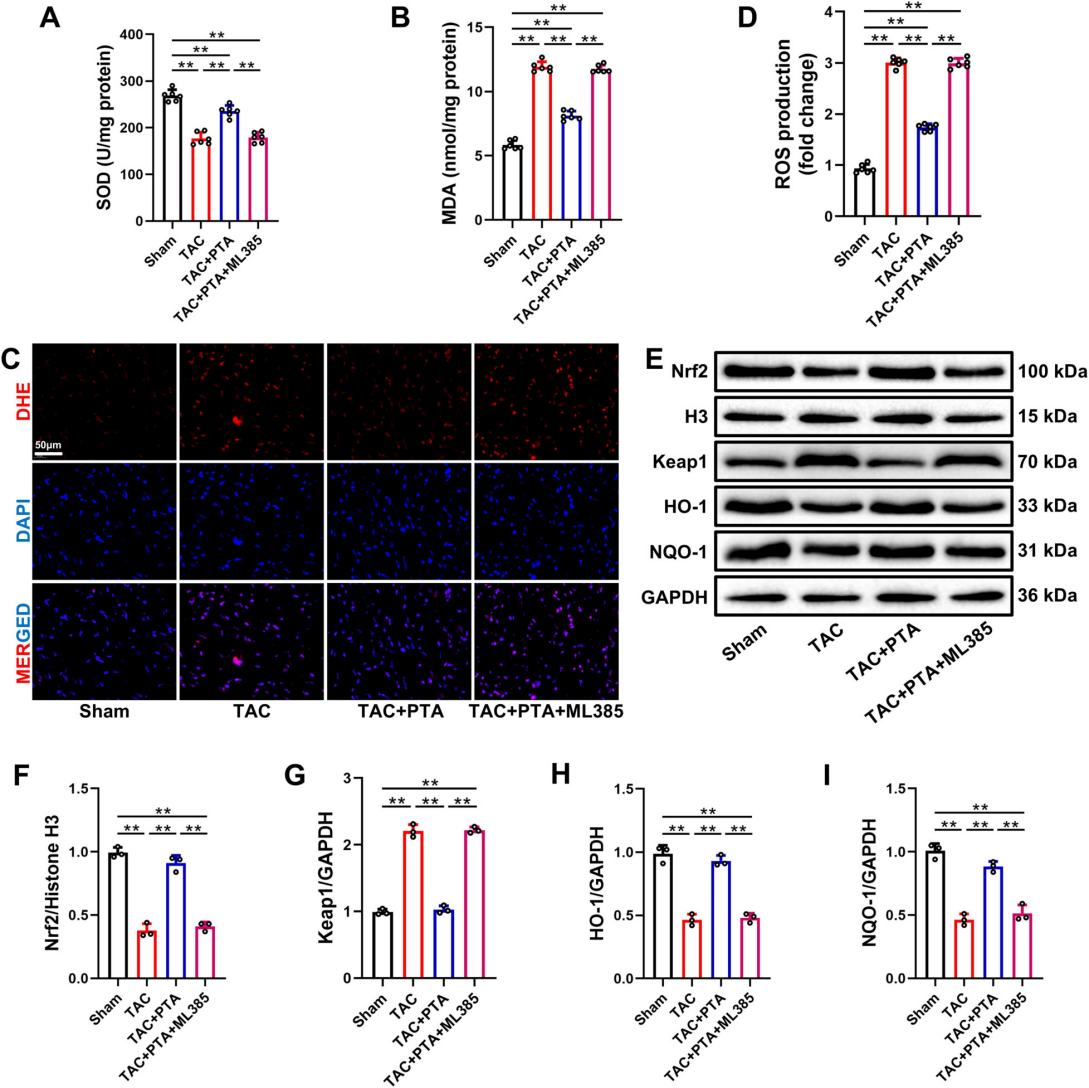
PTA mitigates myocardial oxidative stress in mice after TAC surgery by activating the Nrf2 signaling pathway. **(A)** SOD activities in the heart tissues (*n* = 6 mice per group). **(B)** MDA contents in the heart tissues (*n* = 6 mice per group). **(C,D)** Representative images of DHE staining and relative ROS level in the heart tissues (*n* = 6 mice per group, scale bar = 50 μm). Red fluorescence corresponds to ethidium oxide, which is formed when dihydroethidium (DHE) enters cells and is oxidized by ROS. Its fluorescence intensity is proportional to the level of ROS. Blue fluorescence represents nuclear staining. **(E–I)** Representative western blot and semi-quantification of Keap1, Nrf2, HO-1 and NQO-1 protein expressions in the heart tissues (*n* = 3 mice per group). PTA was administrated at a concentration of 20 mg/kg. **P < 0.01 between the two indicated groups. The data are presented as the mean ± SEM.

Oxidative stress injury is a critical contributor to the structural and functional deterioration of the myocardium resulting from pressure overload (16, 17). As depicted in Figures 4A,B, compared to the sham group, a significant reduction in SOD activity and a notable elevation in MDA content were observed in the myocardial tissues of mice 4 weeks post-TAC surgery. Furthermore, DHE staining demonstrated a marked increase in ROS production within the myocardium of mice in the TAC group (Figures 4C,D). Importantly, treatment with PTA not only enhanced SOD activity but also reduced the generation of MDA and ROS in the myocardial tissues of TAC + PTA mice. However, the administration of ML385 abolished the protective effects of PTA against myocardial oxidative stress injury induced by TAC operation.

Under various pathological conditions associated with oxidative stress, the activation of the Nrf2 signaling pathway exhibits significant cytoprotective effects (30). Western blot analysis of Nrf2-related molecular expressions disclosed a notable reduction in the nuclear translocation of Nrf2, along with decreases in the expressions of HO-1 and NQO-1, which are downstream effectors of Nrf2, in the myocardial tissue of mice in the TAC group (Figures 4E–I). Additionally, the expression Keap1, a negative regulator of Nrf2, was notably increased after TAC surgery (Figures 4G). Significantly, treatment with PTA inhibited the expression of Keap1, facilitated the nuclear translocation of Nrf2 and upregulated the expressions of HO-1 and NQO-1 in the cardiac tissues of mice in the TAC + PTA group. In contrast, specific inhibition of the Nrf2 signaling pathway abrogated the activating effect of PTA on myocardial Nrf2 signaling in mice following TAC surgery.

### 3.4 PTA inhibits cardiomyocyte pyroptosis in mice after TAC surgery by activating the Nrf2 signaling pathway

**Figure 5:**
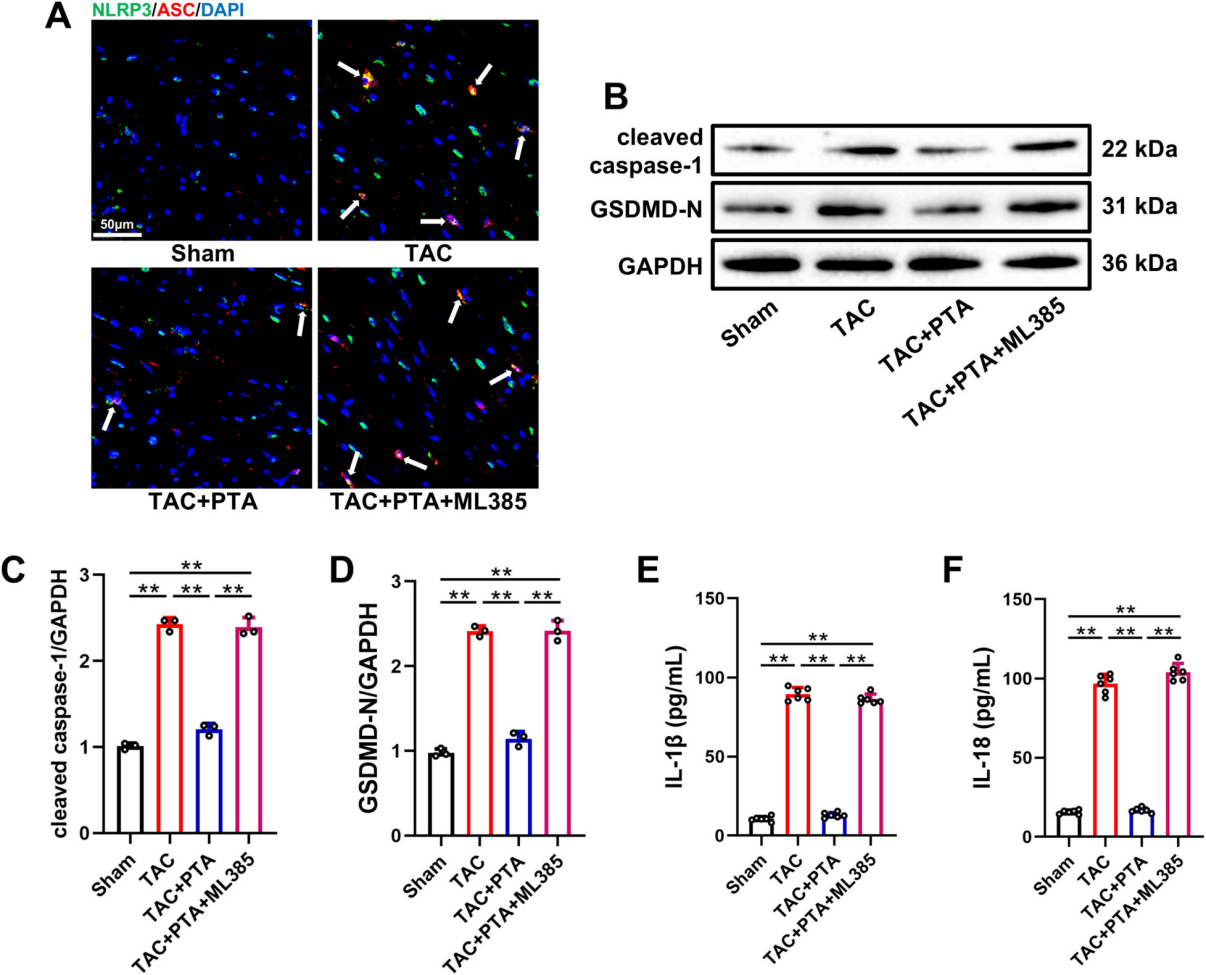
PTA inhibits cardiomyocyte pyroptosis in mice after TAC surgery by activating the Nrf2 signaling pathway. **(A)** Representative images of immunofluorescent staining of NLRP3 inflammasome (scale bar = 50 μm). The white arrows indicate the inflammasomes marked by the co-localization of NLRP3 (green) and ASC (red), and the nucleus were stained with DAPI (blue). **(B–D)** Representative western blot and semi-quantification of cleaved caspase-1 and GSDMD-N protein expressions in the heart tissues (*n* = 3 mice per group). **(E,F)** Concentrations of IL-1β and IL-18 in serum (*n* = 6 mice per group). PTA was administrated at a concentration of 20 mg/kg. **P < 0.01 between the two indicated groups. The data are presented as the mean ± SEM.

Previous studies have reported that cardiomyocyte pyroptosis acts as a crucial factor in myocardial injury induced by pressure overload (31–33). It is well-established that the typical manifestation of pyroptosis is frequently accompanied by the assembly of the NLRP3 inflammasome (34). As illustrated in Figure 5A, immunofluorescence staining demonstrated a notable increase in inflammasome formation, as evidenced by the co-localization of NLRP3 and ASC (indicated by white arrows), in the myocardial tissues of mice in the TAC group. Western blot analysis confirmed that the expression levels of cleaved caspase-1 and GSDMD-N, which are key proteins involved in pyroptosis, were also elevated in the myocardium of TAC mice (Figures 5B–D). Additionally, ELISA assays further measured the elevated levels of IL-1β and IL-18 in the serum, which are the hallmark inflammatory cytokines released through pyroptotic pores, providing functional evidence of the downstream consequences of pyroptosis (Figures 5E,F). Of note, PTA treatment suppressed the formation of the NLRP3 inflammasome, inhibited the expressions of cleaved caspase-1 and GSDMD-N and reduced the serum levels of IL-1β and IL-18. As expected, ML385 administration abrogated the inhibitory effect of PTA on myocardial pyroptosis in mice after TAC surgery.

### 3.5 PTA exhibits the anti-hypertrophic and anti-oxidative effects in Ang Ⅱ-treated NMCMs by activating the Nrf2 signaling pathway

**Figure 6:**
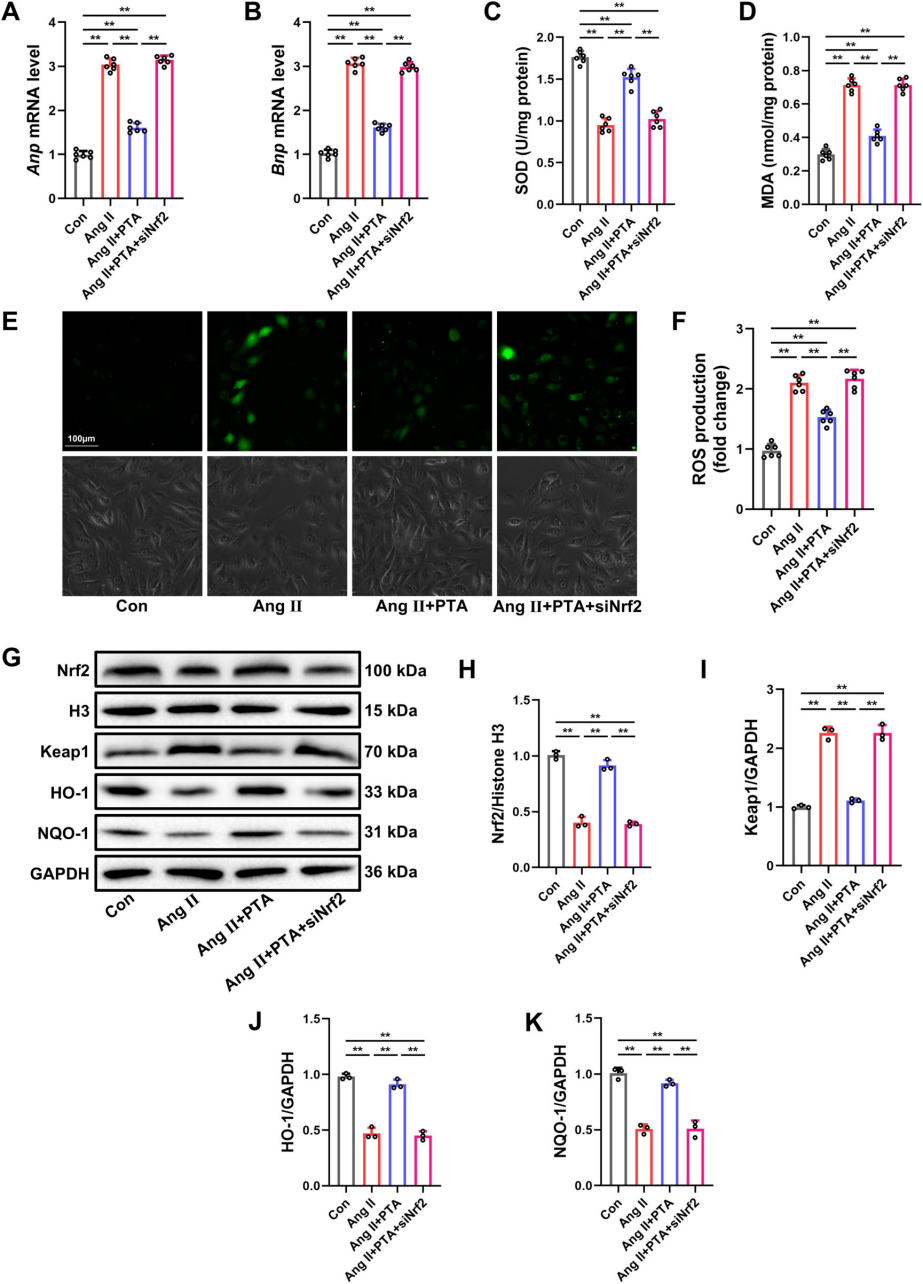
PTA exhibits the anti-hypertrophic and anti-oxidative effects in Ang Ⅱ-treated NMCMs by activating the Nrf2 signaling pathway. **(A,B)** qRT-PCR analysis of *Anp* and *Bnp* mRNA levels in NMCMs (*n* = 6 samples per group). **(C)** SOD activities in NMCMs (*n* = 6 samples per group). **(D)** MDA contents in NMCMs (*n* = 6 samples per group). **(E,F)** Representative images of DCFH-DA staining and relative ROS level in NMCMs (*n* = 6 samples per group, scale bar = 100 μm). Red fluorescence corresponds to 2′,7′-dichlorodihydrofluorescein (DCF), which is generated by the ROS-mediated oxidation of DCFH-DA after it enters cells. Its fluorescence intensity is proportional to the intracellular ROS level. **(G–K)** Representative western blot and semi-quantification of Keap1, Nrf2, HO-1 and NQO-1 protein expressions in NMCMs (*n* = 3 samples per group). PTA was administrated at a concentration of 100 µM. **P < 0.01 between the two indicated groups. The data are presented as the mean ± SEM.

To elucidate the impact and underlying molecular mechanisms of PTA on myocardial injury induced by pressure overload in an *in vitro* setting, we investigated the cytoprotective effects of PTA in Ang Ⅱ-treated NMCMs. As depicted in Figures 6A,B, treatment with Ang Ⅱ for 48 h led to a significant increase in the mRNA levels of ANP and BNP in NMCMs, thereby confirming the successful establishment of the *in vitro* model. Further assessment of cellular oxidative stress levels demonstrated that, in comparison with the Con group, Ang Ⅱ-treated NMCMs exhibited reduced activity of SOD and elevated levels of MDA and ROS (Figures 6C–F). Of note, PTA treatment substantially mitigated the oxidative stress injury triggered by Ang Ⅱ in NMCMs. Moreover, Ang Ⅱ-induced the increased expression of Keap1, the suppression of nuclear translocation of Nrf2 and the decreased expressions of HO-1 and NQO-1 were all reversed following PTA administration (Figures 6G–K). In line with the *in vivo* findings, the employment of Nrf2 siRNA to inhibit Nrf2 expression not only counteracted the stimulatory effect of PTA on the Nrf2 signaling pathway, but also abrogated the anti-hypertrophic and anti-oxidative effects of PTA in Ang Ⅱ-induced NMCMs. Collectively, these results validate that PTA effectively reduced the susceptibility of Ang Ⅱ-treated cardiomyocytes to oxidative stress *in vitro* via the activation of the Nrf2 signaling pathway.

### 3.6 PTA shows the anti-pyroptosis effect in Ang Ⅱ-treated NMCMs by activating the Nrf2 signaling pathway

**Figure 7:**
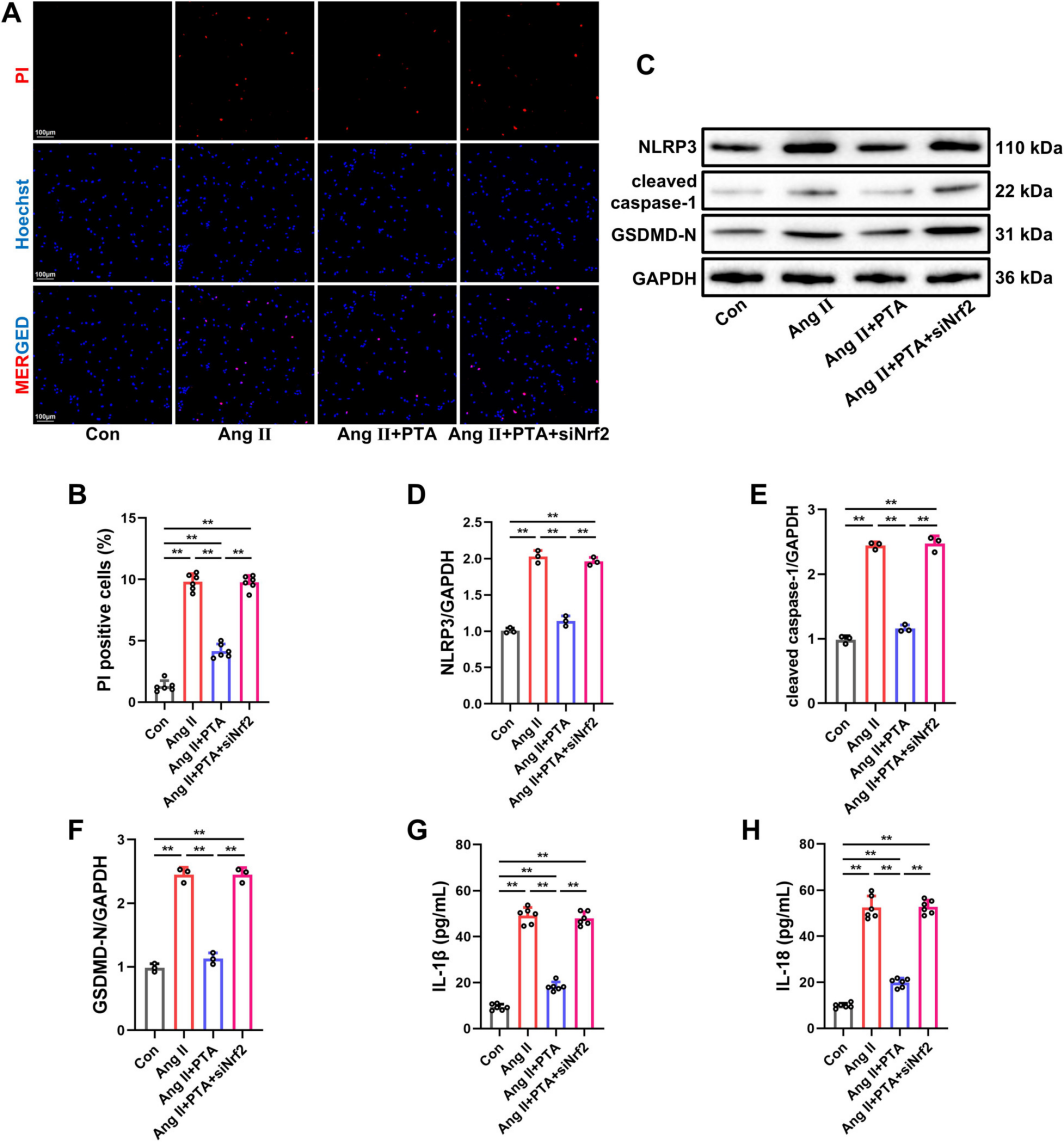
PTA shows the anti-pyroptosis effect in Ang Ⅱ-treated NMCMs by activating the Nrf2 signaling pathway. **(A,B)** Representative images of PI staining and quantification analysis of PI-positive NMCMs (*n* = 6 samples per group, scale bar = 100 μm). Red fluorescence indicates the staining of propidium iodide (PI), which binds to DNA within the nuclei of pyroptotic cells. Blue fluorescence represents nuclear staining. **(C–F)** Representative western blot and semi-quantification of NLRP3, ASC, cleaved caspase-1 and GSDMD-N protein expressions in NMCMs (*n* = 3 samples per group). **(G,H)** Concentrations of IL-1β and IL-18 in cell supernatant (*n* = 6 samples per group). PTA was administrated at a concentration of 100 µM. **P < 0.01 between the two indicated groups. The data are presented as the mean ± SEM.

Subsequently, we also investigated the effects of PTA on Ang Ⅱ-induced pyroptosis in NMCMs at the cellular level. As shown in Figures 7A,B, compared to the Con group, the number of PI-stained positive NMCMs was increased in the Ang Ⅱ group, while PTA treatment significantly reduced Ang Ⅱ-induced cardiomyocyte death. Western blot analysis further disclosed that PTA treatment markedly suppressed the expressions of inflammasome-forming proteins, namely NLRP3 and ASC, as well as the expression of pro-pyroptosis proteins, including cleaved caspase-1 and GSDMD-N, in Ang Ⅱ-treated NMCMs (Figures 7C–F). Additionally, the occurrence of pyroptosis facilitates the activation of pro-inflammatory factors, including IL-18 and IL-1β, and the results of ELISA tests revealed that PTA remarkably ameliorated the increased levels of IL-1β and IL-18 induced by Ang Ⅱ in cell supernatant (Figures 7G,H). Of particular interest, transfection with Nrf2 siRNA significantly abolished the anti-pyroptotic effects of PTA in Ang Ⅱ-treated NMCMs. These results provide further evidence that the protective effect of PTA against pressure overload-induced pyroptosis in cardiomyocytes is mediated through the activation of the Nrf2 signaling pathway.

## 4 Discussion

Inhibition of pathological cardiac hypertrophy represents a crucial strategy for mitigating HF triggered by pressure overload (35, 36). However, current clinical therapies primarily focus on alleviating the clinical symptoms of HF patients (7). Consequently, it is of utmost significance to explore novel and effective pharmacological agents for the treatment of pathological cardiac hypertrophy, thereby delaying or reversing the progression of HF. Extensive preclinical research has demonstrated that PTA, an active component extracted from the traditional Chinese medicine *Caesalpinia sappan*, exhibits multifaceted cardioprotective effects against doxorubicin-induced cardiotoxicity, myocardial ischemia-reperfusion injury and autoimmune myocarditis (12, 13). These findings suggest that PTA holds substantial therapeutic potential in the realm of cardioprotection. Nevertheless, the role of PTA in pathological cardiac hypertrophy has not been investigated yet. Our study is the first to demonstrate that PTA protects against pressure overload-induced cardiac injury and dysfunction. Moreover, the protective efficacy of PTA in suppressing oxidative stress and pyroptosis further substantiates its potential practical and clinical utility in the management of HF.

The heart's principal function is to sustain perfusion in peripheral organs, thereby fulfilling their oxygen and nutrient requirements under both physiological and stress conditions. In order to achieve this objective, particularly when confronted with augmented preload and afterload, the heart frequently undergoes enlargement, a condition referred to as hypertrophy (37). Initially, cardiac hypertrophy represents an adaptive response to hemodynamic overload, intended to enhance cardiac contractility. Nevertheless, under persistent stimulus of abnormal hemodynamic stress caused by cardiovascular diseases such as hypertension and myocardial infarction, this adaptive hypertrophy may transition into pathological cardiac hypertrophy via cardiac remodeling, potentially progressing to heart failure (38).

A key characteristic of cardiac remodeling triggered by pressure overload is cardiomyocyte hypertrophy, whereas the augmentation of myocardial interstitial fibrosis constitutes another pivotal feature of pathological cardiac remodeling (28, 29). It is noteworthy that the progression of cardiac remodeling inevitably leads to the deterioration of heart function (39). Our study initially demonstrated that PTA treatment significantly improved cardiac contractility in mice subjected to TAC surgery. Further investigation into structural changes revealed that PTA treatment notably reduced the mean cross-sectional area of cardiomyocytes, decreased heart mass, and attenuated ventricular wall thickness. Additionally, the expressions of ANP and BNP in both the myocardium of TAC-treated mice and in cardiomyocytes exposed to Ang II were markedly suppressed by PTA administration. Furthermore, findings from myocardial fibrosis analysis showed that PTA treatment remarkably decreased fibrous collagen deposition and suppressed the expression of pro-fibrotic genes.

Persistent oxidative stress injury serves as a pivotal mechanism mediating the transition from compensatory cardiac hypertrophy to heart failure (15). It has been well-documented that prolonged exposure of the hypertrophied heart to diverse pathological stimuli fosters the production of ROS (40). The resultant elevated levels of ROS overwhelm the body's antioxidant capacity, culminating in oxidative stress injury to the myocardium. Consequently, this process significantly promotes the development and progression of maladaptive cardiac remodeling and cardiac dysfunction (16, 17). Importantly, prior research has established that PTA exhibits remarkable antioxidant properties. Zeng and colleagues demonstrated that PTA mitigated lipopolysaccharide-induced brain immune and neuroinflammation by suppressing oxidative and nitrative stress (41). Huang et al. reported that PTA inhibited osteoclastogenesis by reducing oxidative stress in RAW264.7 cells (42). Cui and colleagues discovered that PTA suppressed ROS production in DOX-induced cardiotoxicity and myocardial ischemia-reperfusion injury (12). In line with these findings, our study revealed that PTA treatment reversed the pressure overload-induced increase in ROS and MDA accumulation, as well as the decrease in SOD activity, both *in vivo* and *in vitro*.

Given that Nrf2 is a key molecule in the antioxidant defense system, and activation of the Nrf2 signaling pathway has been shown to alleviate oxidative stress by inhibiting the generation of ROS, thereby conferring protection against various cardiovascular diseases (23, 24), we endeavored to conduct a more in-depth exploration into whether the Nrf2 signaling pathway mediates the antioxidant effects of PTA in the context of pathological cardiac hypertrophy. Results from our research revealed that, in the myocardium of mice following TAC operation and in NMCMs treated with Ang II, PTA not only promoted the nuclear translocation of Nrf2, but also upregulated the expression of downstream molecules (HO-1 and NQO-1). Whereas, administration of ML385 to block the Nrf2 signaling pathway or Nrf2 siRNA to inhibit Nrf2 expression not only counteracted the alleviating effect of PTA on oxidative stress injury but also abrogated the amelioration action of PTA on cardiac remodeling and dysfunction. These results indicate that the protective effect of PTA on pathological cardiac hypertrophy is achieved through the activation of the Nrf2 signaling pathway.

Growing evidence suggests that oxidative stress induced by the imbalance of ROS homeostasis has been identified as a major contributor to cardiomyocyte death in multiple cardiovascular diseases (19, 43). Recent studies have revealed that ROS is a key trigger for the occurrence of pyroptosis through the activation of NLRP3 inflammasome (18, 19). On one hand, pyroptosis leads to irreversible loss of cardiomyocytes, and on the other hand, pyroptosis induces and exacerbates the inflammatory response in myocardial tissue, jointly playing a pivotal role in the pathogenesis of pathological cardiac hypertrophy (21, 44). Therefore, targeted interventions against ROS generation and the resultant cardiomyocyte pyroptosis under pathological conditions may offer novel therapeutic strategies for HF induced by pressure overload. Our findings demonstrate that TAC surgery led to significant activation of the NLRP3 inflammasome and marked upregulation of pyroptosis in the myocardium of mice. Conversely, treatment with PTA effectively suppressed cardiomyocyte pyroptosis and the release of pro-inflammatory cytokines (IL-18 and IL-1β). Of note, various molecular mechanisms linking oxidative stress, inflammasome, pyroptosis, and the progression of cardiac pathology have been documented, with Nrf2 emerging as a pivotal player (45), highlighting the potential application of Nrf2 inducers as cardioprotective compounds in preventing pyroptosis. Our study further found that treatment with ML385 or Nrf2 siRNA abolished the inhibitory effect of PTA on cardiomyocyte pyroptosis, indicating that PTA exerts its protective effect against pathological cardiac hypertrophy by activating the Nrf2 signaling pathway to suppress NLRP3 inflammasome activation and pyroptosis.

## 5 Conclusion

The results of our research indicate that PTA treatment alleviates cardiac hypertrophy and retards myocardial fibrosis, thereby protecting against pressure overload-induced cardiac remodeling and dysfunction. Mechanistically, PTA exerts these cardioprotective effects through activation of the Nrf2 signaling pathway, which mitigates oxidative stress in myocardial tissue and inhibits NLRP3 inflammasome-mediated cardiomyocyte pyroptosis. In summary, these discoveries contribute to our understanding of PTA as a potential therapeutic option for pathological cardiac hypertrophy induced by pressure overload and shed light on the underlying mechanisms involved, providing robust evidence for future clinical application of PTA in the treatment of HF and other cardiovascular diseases.

## Data Availability

The raw data supporting the conclusions of this article will be made available by the authors, without undue reservation.

## Supplementary material

Supplementary Figure S1 PTA alleviated cardiac dysfunction and hypertrophy in mice after TAC surgery in a dose-dependent manner. (A-D) Quantitative analysis of LVEF, LVFS, IVSd and LVPWd (*n* = 6 mice per group). (E) The HW/BW ratio (*n* = 6 mice per group). (F) The HW/TL ratio (*n* = 6 mice per group). PTA was administrated at a concentration of 10 mg/kg or 20 mg/kg. ***P* < 0.01 between the two indicated groups. The data are presented as the mean ± SEM.

Supplementary Figure S2 PTA alleviated oxidative stress and inflammatory injury in Ang II-treated NMCMs in a dose-dependent manner. (A) SOD activities in NMCMs (*n* = 6 samples per group). (B) MDA contents in NMCMs (*n* = 6 samples per group). (C-D) Concentrations of IL-1β and IL-18 in cell supernatant (*n* = 6 samples per group). PTA was administrated at a concentration of 50 µM or 100 µM. ***P* < 0.01 between the two indicated groups. The data are presented as the mean ± SEM.

Supplementary Figure S3 Nrf2 siRNA significantly reduced the transcriptional and translational levels of Nrf2 in cardiomyocytes. (A) Representative western blot. (B) Semi-quantification of Nrf2 protein expression in NMCMs (*n* = 3 samples per group). (C) qRT-PCR analysis of *Nrf2* mRNA level in NMCMs (*n* = 6 mice per group). ***P* < 0.01 between the two indicated groups. The data are presented as the mean ± SEM.
