# Supplementary figures and images for: Protosappanin A protects against pathological cardiac hypertrophy by inhibiting oxidative stress and NLRP3 inflammasome-mediated pyroptosis via activation of the Nrf2 signaling pathway
Source: Front Cardiovasc Med. 2026 Jan 9;12:1682641. doi: 10.3389/fcvm.2025.1682641 (PMC12827537; doi:10.3389/fcvm.2025.1682641)

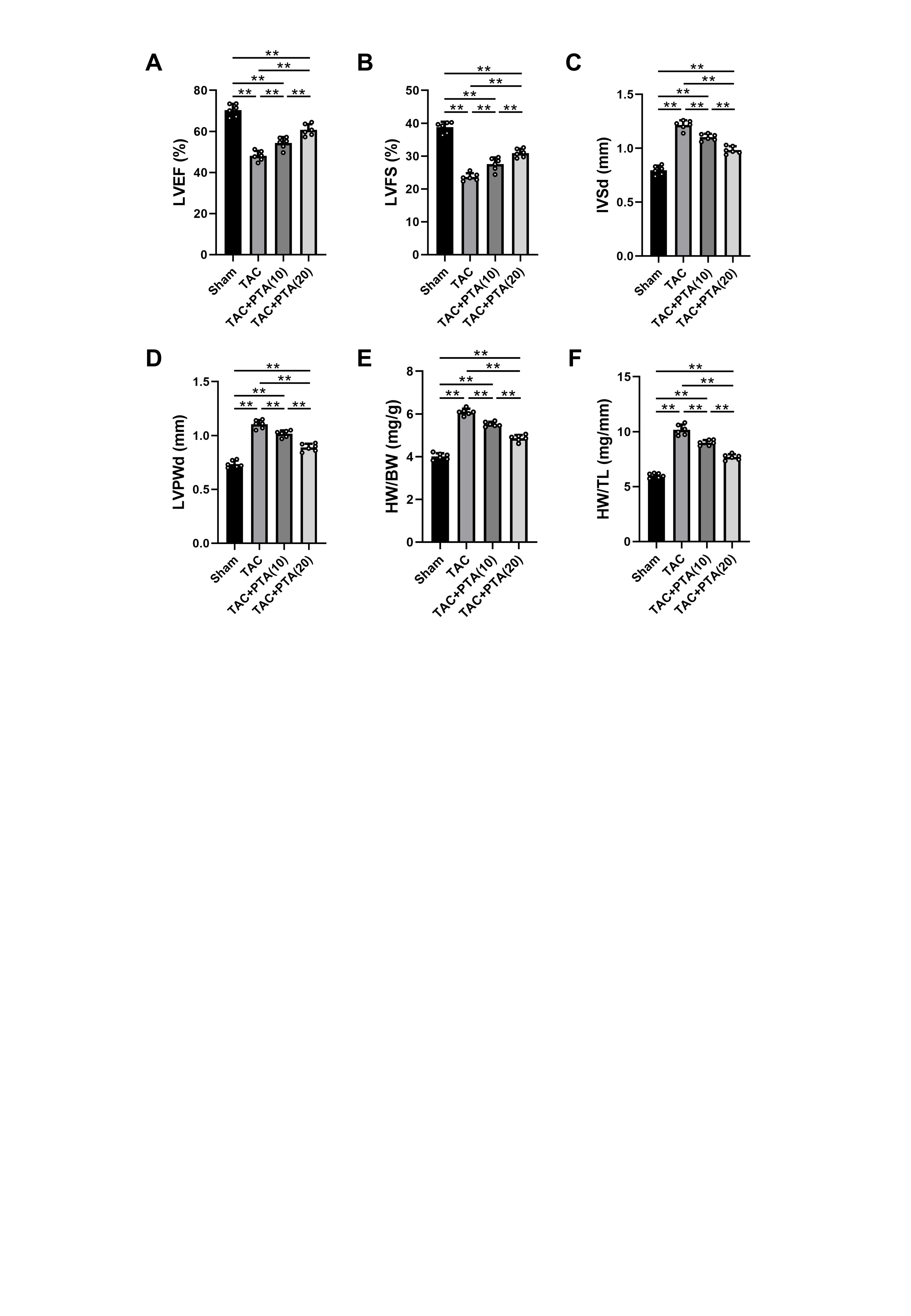

Supplement: Supplementary Figure S1 — PTA alleviated cardiac dysfunction and hypertrophy in mice after TAC surgery in a dose-dependent manner. (A-D) Quantitative analysis of LVEF, LVFS, IVSd and LVPWd (n = 6 mice per group). (E) The HW/BW ratio (n = 6 mice per group). (F) The HW/TL ratio (n = 6 mice per group). PTA was administrated at a concentration of 10 mg/kg or 20 mg/kg. **P < 0.01 between the two indicated groups. The data are presented as the mean ± SEM. [file Image1.tif]

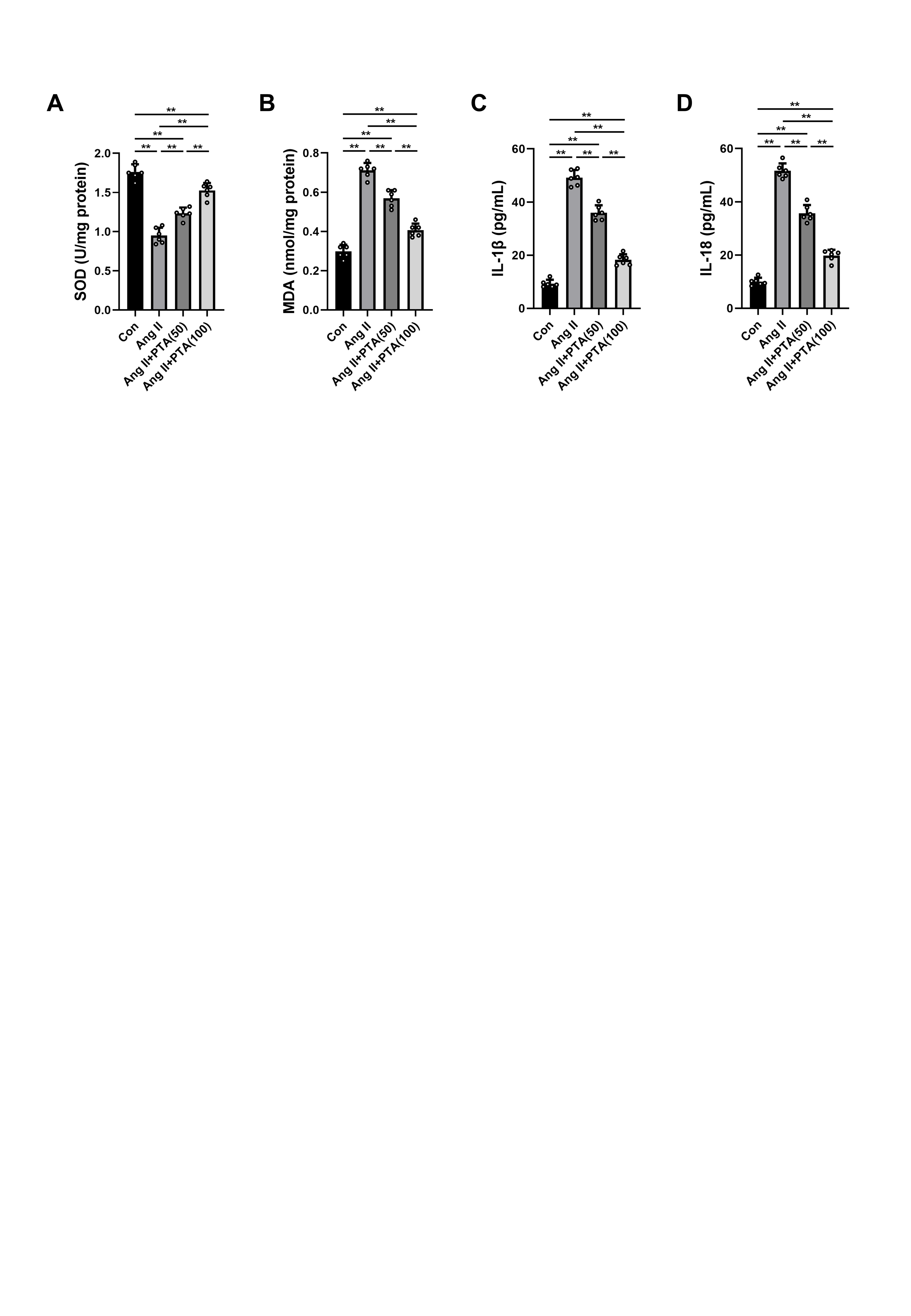

Supplement: Supplementary Figure S2 — PTA alleviated oxidative stress and inflammatory injury in Ang II-treated NMCMs in a dose-dependent manner. (A) SOD activities in NMCMs (n = 6 samples per group). (B) MDA contents in NMCMs (n = 6 samples per group). (C-D) Concentrations of IL-1β and IL-18 in cell supernatant (n = 6 samples per group). PTA was administrated at a concentration of 50 µM or 100 µM. **P < 0.01 between the two indicated groups. The data are presented as the mean ± SEM. [file Image2.tif]

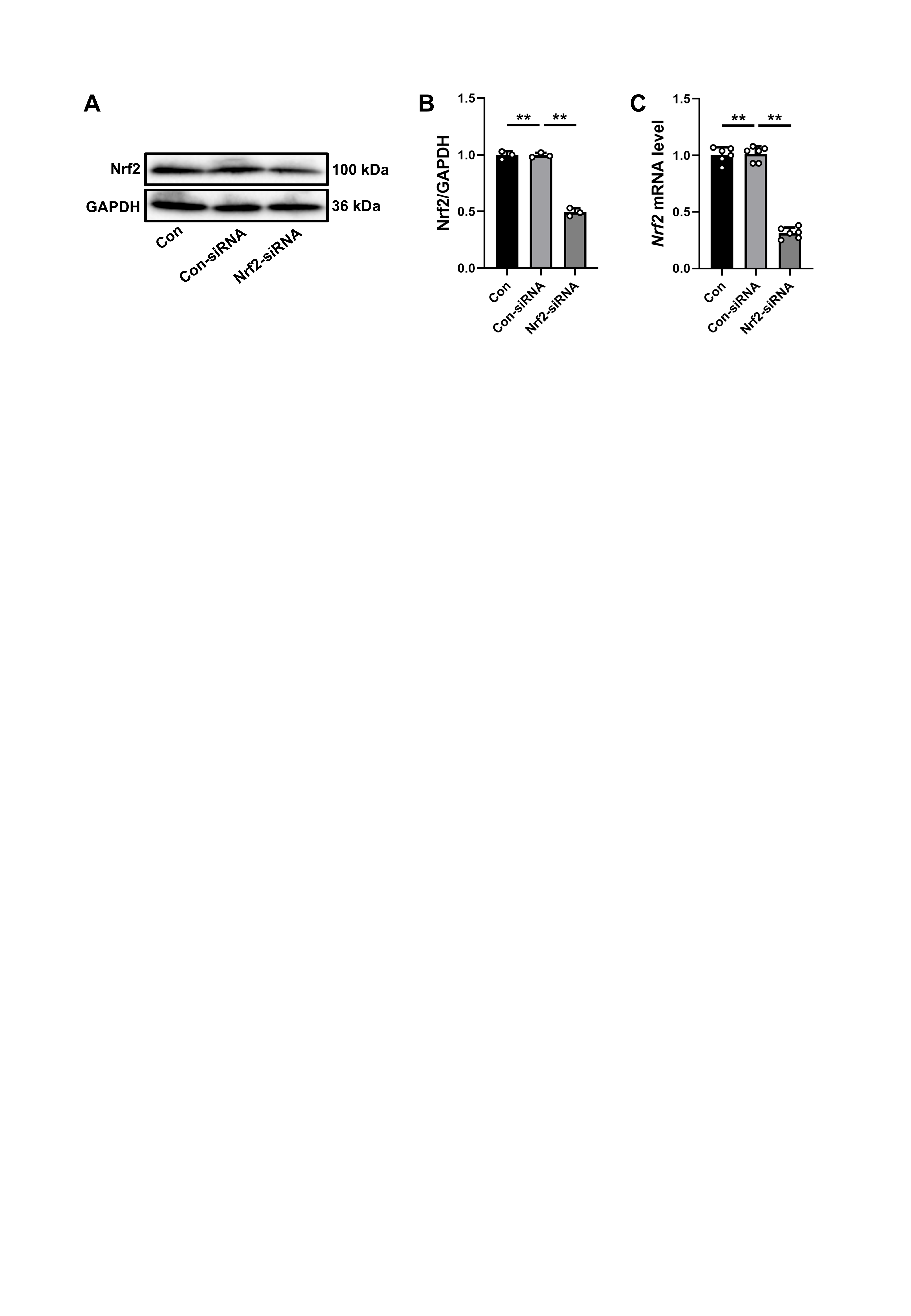

Supplement: Supplementary Figure S3 — Nrf2 siRNA significantly reduced the transcriptional and translational levels of Nrf2 in cardiomyocytes. (A) Representative western blot. (B) Semi-quantification of Nrf2 protein expression in NMCMs (n = 3 samples per group). (C) qRT-PCR analysis of Nrf2 mRNA level in NMCMs (n = 6 mice per group). **P < 0.01 between the two indicated groups. The data are presented as the mean ± SEM. [file Image3.tif]
